# A Giant Borderline Phyllodes Tumor of Breast With Skin Ulceration Leading to Non-Insular Tumorigenic Hypoglycemia: A Case Report and Literature Review

**DOI:** 10.3389/fendo.2021.651568

**Published:** 2021-03-25

**Authors:** Jinlu Zhao, Meizhuo Gao, Yi Ren, Shaodong Cao, He Wang, Ruisheng Ge

**Affiliations:** ^1^ Department of General Surgery, The Fourth Affiliated Hospital of Harbin Medical University, Harbin, China; ^2^ Department of Imaging, The Fourth Affiliated Hospital of Harbin Medical University, Harbin, China; ^3^ Department of Pathology, The Fourth Affiliated Hospital of Harbin Medical University, Harbin, China

**Keywords:** giant borderline phyllodes tumors, breast, skin ulceration, non-islet cell tumor hypoglycemia, insulin-like growth factor-II

## Abstract

Phyllodes tumor (PT) is a special type of breast tumors, including three types: malignant, borderline, and benign. Most of these tumors form unilateral disease and can rapidly increase in size. The occurrence of axillary lymph node metastasis is rare. Tumor-associated hypoglycemia can be divided into non-islet cell tumor and insulinoma. In non-islet cell tumor hypoglycemia (NICTH), a considerable high molecular weight form of insulin like growth factor 2 (IGF-2) is formed, which abnormally binds to insulin receptors in the tissues and causes hypoglycemia. Breast phyllodes tumors with NICTH are rare and first reported in 1983. Surgical resection is the main treatment and hypoglycemia symptoms usually resolve after surgery. Nevertheless, prior to surgery, intravenous glucose infusion is used to maintain blood glucose levels. A female patient presented with a rapidly growing breast mass and was diagnosed with a phyllodes tumor with NICTH at our hospital in August 2020; she was successfully treated through surgical resection. We reviewed the relevant literature to investigate and analyze the relationship between NICTH and phyllodes tumors, as well as optimize its diagnosis and treatment.

## Introduction

Among the various forms of breast tumors, phyllodes tumor (PT) has the lower incidence rate (1/100,000 individuals) (1). Moreover, the total risk of malignancy is approximately 2.1/1 million individuals (2). According to WHO data, this type includes malignant, borderline and benign tumors (3). This is mostly unilateral disease (4), and the probability of axillary lymph node metastasis is extremely low (4, 5). Hypoglycemia is a common endocrine emergency, usually associated with diabetes and endocrine disorders (6). Certain tumors may lead to hypoglycemia which are known as tumor-associated hypoglycemia. Tumor-associated hypoglycemia can be classified into two categories according to the mechanism underlying its development. The most common mechanism is high secretion of insulin by insulinomas. The second mechanism, termed non-islet cell tumor hypoglycemia (NICTH), involves the formation of a certain high molecular weight form of insulin like growth factor 2 (IGF-2), which abnormally binds to insulin receptors in the tissues, leads to increased glucose utilization, and causes hypoglycemia (7). NITCH was first reported on primary hepatocellular carcinoma in 1929 (8). Clinically, breast PTs with NITCH are rare and first reported in 1983 (9). We investigated and detected a type of NICTH initiation method, which is mainly caused by giant borderline PT. Preoperative hypoglycemia may be affected by IGF-2 produced by PTs.

## Case Description

**Figure 1:**
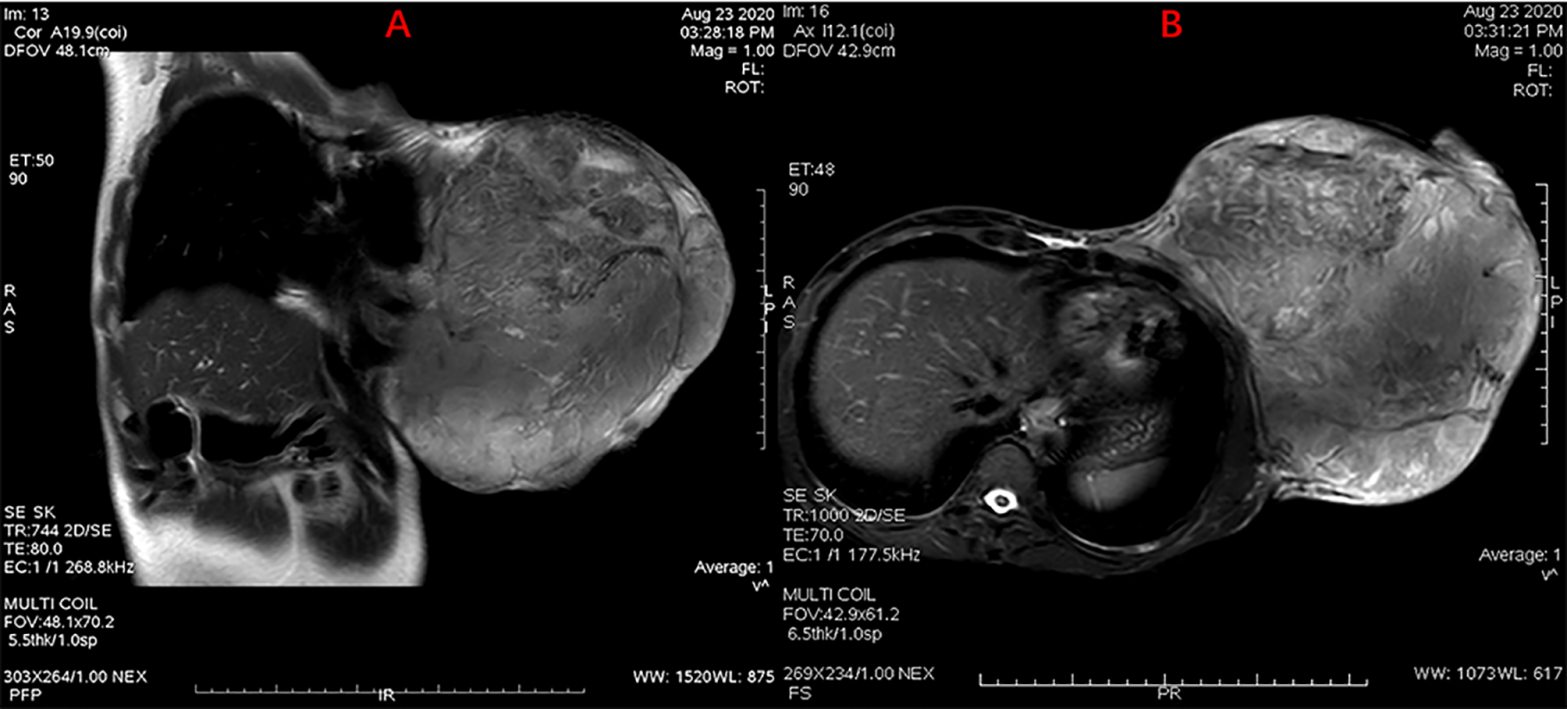
MRI scan showing a giant mass in the left breast with skin ulceration. Without chest wall infiltration or axillary lymph node metastasis were observed. **(A)** Sagittal MR scanning, **(B)** Horizontal MR scanning.

**Figure 2:**
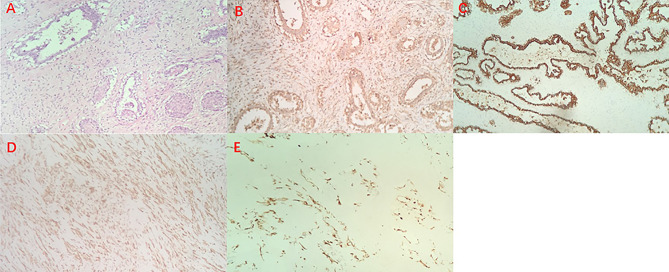
Hematoxylin and eosin staining of the borderline Phyllodes Tumor of Breast (original magnification ×100) **(A)**, Immunohistochemistry staining revealed positive expression of IGF-2 **(B)**, CK **(C)**, SMA **(D)** and Desmin **(E)**.

A female patient aged 45 years was admitted to the hospital with a primary complaint of a left breast mass present for 6 months. The patient was a farmer without family history and didn’t receive other treatment before. The mass increased rapidly in the past 3 months, reaching the size of a basketball with local skin ulceration and infection. The mass (25 cm in diameter) made the breasts asymmetric and was observed in the left breast with tough texture and poor mobility. The surface of the mass is uneven with skin ulceration. Bilateral axillary lymph nodes were not touched. Examination by magnetic resonance imaging showed that the solid mass had not invaded the chest wall muscle (**Figure 1**). On the second and third day of admission, she experienced fasting hypoglycemia associated with fatigue, cold sweats, and confusion. Blood examination showed severe hypoglycemia (0.78 mmol/L), hypoinsulinemia (<1.39 pmol/L), and C peptide level (0.01 nmol/L). The serum insulin antibody was negative and the patient didn’t take sulfonylureas previously. The level of carcinoembryonic antigen (CEA), alpha fetoprotein(AFP) and carbohydrate antigen153 (CA153) were within the normal range. The unenhanced magnetic resonance scan showed a regular pancreas and liver contour. Hence, the presence of islet cell tumor and hepatocellular carcinoma (hepatocellular carcinoma often lead to NICTH) were ruled out, and symptomatic treatments (e.g., intravenous infusion of 10% glucose solution) were administered to regulate the concentration of blood glucose. After symptomatic treatments, the symptoms above completely relieved. A comprehensive assessment of patient signs, clinical manifestations, medical history, auxiliary examinations, and preoperative puncture pathology considered the possibility of PTs. On August 27, 2020, the patient underwent left mastectomy. Intraoperative frozen section diagnosis revealed a fibroepithelial tumor, and the possibility of PT was not excluded. The upper, lower, inner, and outer edges of skin samples were submitted for examination, and there were no tumor cells found. The breast incision flap was sutured without axillary lymph node dissection. Immunohistochemical stains with antibodies against IGF-2 (IGF-2 Rabbit pAb A2086, dilution 1:300), CKpan (RAB-0050, dilution 1:200), SMA (alpha smooth muscle actin Rabbit pAb A7248, dilution 1:200) and Desmin (DES Rabbit pAb A0699, dilution 1:200) were performed. Postoperative immunohistochemical staining results (**Figure 2**) were as follows: IGF-2(+), CKpan (+), SMA (+), Desmin (+). Postoperative paraffin pathology analysis (left breast) showed that the mass was borderline PT, part of the epithelium was squamous, and surface skin ulcers were formed (tumor diameter: 25 cm). The patient recovered well after the operation, and blood glucose and insulin levels returned to normal. No adjuvant therapy was administered after surgery and there was no tumor recurrence or metastasis detected at 3-month follow-up. The patient’s family has signed the written consent.

## Discussion

Typically, borderline or malignant PT do not involve multiple lesions, and most of them form unilateral disease. The total tumor size of surgical specimens ranges from no residue after biopsy to 38 cm, while the size of PT is larger for malignant PT (4). This patient had a unilateral giant borderline PT; the tumor grew rapidly within 3 months, which made the skin of the left breast tight and thin. The huge mass caused ischemia and formed ischemic ulcers. In addition, the patient developed severe hypoglycemia prior to surgery. Pancreatic magnetic resonance imaging and serological examinations ruled out the possibility of insulinomas. One day after surgery, the blood glucose and insulin levels of the patient rapidly returned to normal. Postoperative immunohistochemical staining results were as follows: IGF-2(+), CKpan (+), SMA (+), Desmin (+). Thus, the patient’s preoperative hypoglycemia may have been non-islet cell tumor hypoglycemia (NICTH).

A study analyzed IGF-2 of 44 patients with NICTH, found that 31 patients had high levels of big IGF-2. Furthermore, the study was performed to determine the presence of IGF-2 in the tumors of 20 NICTH patients. Of those, high levels of big IGF-2 were found in the serum of 18 patients (10). These data indicate that hypoglycemia is affected by high molecular weight IGF-2, leading to the development of NITCH. The symptoms of preoperative hypoglycemia in this case may have been caused by IGF-2 in tumor cells.

In addition, another study had found (11) that IGF-2 played a certain role in the diagnosis of the large breast borderline PTs with hypoglycemia. IGF-2 was detected in the tumor and corresponding normal tissues and the measured high serum IGF-2 level led to a preoperative diagnosis. Following mastectomy, the postoperative levels of IGF-2 in the serum gradually returned to normal. Masahiro Hikichi et al. (7) measured the high molecular weight IGF-2 in the serum and tumor tissues of a giant borderline PTs with hypoglycemia through western blotting and immunohistochemical analysis. The western blotting showed that significant levels of high molecular weight IGF-2 were accumulated in tumors at the preoperative stage, but not in the serum obtained 3 days after tumor resection. This confirmed that the core factor of hypoglycemia is caused by the high molecular weight IGF-2. Therefore, for patients with breast PTs with severe hypoglycemia in whom high levels of IGF-2 are detected in tumor cells or serum, the diagnosis of NICTH can be considered. Nevertheless, Jannin A et al. (12) considered that an IGF-2/IGF-1 ratio > 10 was much more useful than the measurement of Big IGF-2, they measured the IGF-2/IGF-1 ratio in six patients with NICTH, found that all of the ratio are more than 10 and the median is 31.8. Unfortunately, we were unable to detect the level of IGF-2 and IGF-1 in serum before and after surgery in our study. However, we excluded the possibility of islet cell tumor by the preoperative examination. According to the perioperative blood glucose changes, the postoperative immunohistochemical staining results and the relevant literature reports. We inferred that preoperative hypoglycemia was most likely caused by the high expression of IGF-2 in the phyllodes tumor. It provides experience for the diagnosis of NICTH.

The first-choice treatment of breast PT is surgical resection (13). Without adjuvant treatment, patients with borderline and malignant PT do not experience recurrence after local tissue resection, and the two conditions are similar (14). The PTs that caused NITCH will no longer produce IGF-2 after surgical resection, the symptoms of NICTH will resolve, and blood glucose levels will return to normal (7, 12, 15–17). Of note, hypoglycemia caused by NICHT is serious, and intravenous glucose infusion is used prior to surgery to regulate blood glucose levels (7, 12, 15–17). Surgical resection requires negative margins. Many studies have confirmed this view. A study of 164 cases of PTs (18) showed that the length of the margin is not associated with local recurrence, whereas a positive margin is related to recurrence. Another study of 183 cases of PTs (19) also suggested that the size of the surgical margin is not linked to local recurrence. Noordman PCW et al. (13) stated that the primary treatment for borderline and malignant PTs is extensive local excision with tumor-negative resection margins. In addition, if the skin cannot be preserved, it can be filled with the method of rectus abdominis musculocutaneous or latissimus dorsi, which is important for reconstruction after mastectomy (20). Considering that the risk of axillary lymph node metastasis is extremely low, routine lymph node dissection is not recommended (4, 5). In our present case, there was no axillary lymph node enlargement found in the preoperative imaging. Therefore, this patient did not undergo axillary lymph node dissection during left breast resection, and the results of the intraoperative frozen section diagnosis showed negative margins. As a result, the patient recovered well after operation and no recurrence occurred during 3 months follow-up, which proved our treatment was effective for PTs with NICTH.

## Conclusion

This report presents a case of NITCH caused by a giant borderline PT of the breast. However, it was a pity that we were unable to measure the serum IGF-2 and IGF-1 levels before and after surgery. After excluding the possibility of islet cell tumor by the preoperative examination. And then combining with relevant literature and perioperative blood glucose changes and the postoperative immunohistochemical staining, we inferred that the hypoglycemia symptoms were most likely caused by the high expression of IGF-2 in the phyllodes tumor. The disease and related clinical manifestations are extremely rare in clinical practice. To avoid missed diagnosis, it is necessary for clinicians to consider that PTs with hypoglycemia may be caused by NICTH, monitor the blood glucose levels during the perioperative period, and select the most appropriate treatment to avoid delays in diagnosis and treatment. This article can provide useful guidelines for clinical practice.

## Data Availability Statement 

The original contributions presented in the study are included in the article/**Supplementary Material**. Further inquiries can be directed to the corresponding authors.

## Ethics Statement

The studies involving human participants were reviewed and approved by Ethics Committee of the Fourth Affiliated Hospital of Harbin Medical University. The patients/participants provided their written informed consent to participate in this study. Written informed consent was obtained from the individual(s) for the publication of any potentially identifiable images or data included in this article.

## Author Contributions

JZ and MG completed the surgery of the patient. JZ had the subject of the case and provided financial support. YR collected relevant literature and wrote the manuscript. JZ reviewed all related literature and revised the manuscript in this study. HW and RG performed postoperative immunohistochemical staining. SC provided the relevant imaging data. All authors contributed to the article and approved the submitted version.

## Funding

This study was supported by the Excellent youth project of the Fourth Affiliated Hospital of Harbin Medical University (Grant No. HYDSYYXQN202006, principal investigator JZ), the youth project of Science and technology innovation project of Heilongjiang Academy of traditional Chinese Medicine (Grant No. ZHY19-080, principal investigator JZ).

## Conflict of Interest

The authors declare that the research was conducted in the absence of any commercial or financial relationships that could be construed as a potential conflict of interest.
