# Supplementary material for: A Giant Borderline Phyllodes Tumor of Breast With Skin Ulceration Leading to Non-Insular Tumorigenic Hypoglycemia: A Case Report and Literature Review
Source: Front Endocrinol (Lausanne). 2021 Mar 25;12:651568. doi: 10.3389/fendo.2021.651568 (PMC8027481; doi:10.3389/fendo.2021.651568)
Supplement: Supplementary file 1 [file DataSheet_1.pdf]

CARE
case report guidelines

CARE Checklist of information to include when writing a case report

(cc) BY-NC-ND

✓

Topic	Item	Checklist item description	Reported on Line
**Title**	**1**	The diagnosis or intervention of primary focus followed by the words “case report”	✓
**Key Words**	**2**	2 to 5 key words that identify diagnoses or interventions in this case report, including "case report"	Line14-15
**Abstract (no references)**	**3a**	Introduction: What is unique about this case and what does it add to the scientific literature?	Line23-24
	**3b**	Main symptoms and/or important clinical findings	Line26-27
	**3c**	The main diagnoses, therapeutic interventions, and outcomes	Line24-28
	**3d**	Conclusion—What is the main “take-away” lesson(s) from this case?	Line28-30
**Introduction**	**4**	One or two paragraphs summarizing why this case is unique (**may include references**)	Line33-49
**Patient Information**	**5a**	De-identified patient specific information	Line52-53
	**5b**	Primary concerns and symptoms of the patient	Line52-56
	**5c**	Medical, family, and psycho-social history including relevant genetic information	No mention
	**5d**	Relevant past interventions with outcomes	No mention
**Clinical Findings**	**6**	Describe significant physical examination (PE) and important clinical findings	Line54-60
**Timeline**	**7**	Historical and current information from this episode of care organized as a timeline	No mention
**Diagnostic Assessment**	**8a**	Diagnostic testing (such as PE, laboratory testing, imaging, surveys).	Line56-60
	**8b**	Diagnostic challenges (such as access to testing, financial, or cultural)	Line58-60
	**8c**	Diagnosis (including other diagnoses considered)	Line59-61,63-64,70-72
	**8d**	Prognosis (such as staging in oncology) where applicable	Line74-75
**Therapeutic Intervention**	**9a**	Types of therapeutic intervention (such as pharmacologic, surgical, preventive, self-care)	Line61-62,64-65
	**9b**	Administration of therapeutic intervention (such as dosage, strength, duration)	Line61-62
	**9c**	Changes in therapeutic intervention (with rationale)	Line58-62
**Follow-up and Outcomes**	**10a**	Clinician and patient-assessed outcomes (if available)	No mention
	**10b**	Important follow-up diagnostic and other test results	Line74-75
	**10c**	Intervention adherence and tolerability (How was this assessed?)	No mention
	**10d**	Adverse and unanticipated events	No mention
**Discussion**	**11a**	A scientific discussion of the strengths AND limitations associated with this case report	Line80-88
	**11b**	Discussion of the relevant medical literature **with references**	Line78-130
	**11c**	The scientific rationale for any conclusions (including assessment of possible causes)	Line78-130
	**11d**	The primary “take-away” lessons of this case report (without references) in a one paragraph conclusion	Line133-138
**Patient Perspective**	**12**	The patient should share their perspective in one to two paragraphs on the treatment(s) they received	No mention
**Informed Consent**	**13**	Did the patient give informed consent? Please provide if requested	Yes ☑ No ☐
